# Results of Arthroscopic Ankle Arthrodesis with Fixation Using Two Parallel Headless Compression Screws in a Heterogenic Group of Patients

**DOI:** 10.2174/1874325001711010037

**Published:** 2017-02-24

**Authors:** Lukas Kolodziej, Boguslaw Sadlik, Sebastian Sokolowski, Andrzej Bohatyrewicz

**Affiliations:** 1 Orthopaedic, Traumatology and Orthopedic Oncology Clinic, Pomeranian Medical University, Szczecin, Poland; 2 Biological Joint Reconstruction Department, St. Luke’s Hospital, Bielsko-Biala, Poland

**Keywords:** Arthroscopic ankle arthrodesis, Ankle arthritis, Fixation, Headless compression screws, Operative treatment, Neuromuscular disorders

## Abstract

**Background:**

As orthopedic surgeons become skilled in ankle arthroscopy technique and evidence -based data is supporting its use, arthroscopic ankle arthrodesis (AAA) will likely continue to increase, but stabilization methods have not been described clearly. We present a technique for two parallel 7.3-mm headless compression screws fixation (HCSs) for AAA in cases of ankle arthritis with different etiology, both traumatic and non-traumatic, including neuromuscular and inflammatory patients.

**Materials and Methods:**

We retrospectively verified 24 consecutive patients (25 ankles) who underwent AAA between 2011 and 2015. The average follow-up was 26 months (range 18 to 52 months). Arthrodesis was performed in 16 patients due to posttraumatic arthritis (in 5 as a sequela of pilon, 6 ankles, 3 tibia fractures, and 2 had arthritis due to chronic instability after lateral ligament injury), in 4 patients due to neuromuscular ankle joint deformities, and in 4 patients due to rheumatoid arthritis.

**Results:**

Fusion occurred in 23 joints (92%) over an average of 12 weeks (range 6 to 18 weeks). Ankle arthrodesis was not achieved in 2 joints (8%), both in post-pilon fracture patients. The correct foot alignment was not achieved in 4 feet (16%). None of the treated patients required hardware removal.

**Conclusion:**

The presented technique was effective in achieving a high fusion rate in a variety of diseases, decreasing intra- and post-operative hardware complications while maintaining adequate bone stability.

## INTRODUCTION

Arthroscopic ankle arthrodesis (AAA) reduces the time to achieve union, complication rates, length of hospital stay, and procedure costs, and increases patient satisfaction compared to open techniques [1, 2]. Arthroscopic procedures give reliable and reproducible results, even in significant deformity [3, 4]. AAA has gained popularity among orthopedic surgeons over the last two decades [5, 6]. Fixation methods to maintain proper foot alignment long enough to achieve solid bone union, without the need for early hardware removal, is still a major concern [7-18]. Here, we present the results of AAA in 24 patients (25 ankles) with fixation by two parallel 7.3-mm mediolateral headless compression screws (HCSs) in different clinical indications.

We describe a detailed three-step surgical technique that helps surgeons avoid struggling with AAA fixation while maintaining the desired foot position. The presented technique is based on the original open arthrodesis method described by Mann *et al.* in 1991 and further modified [12, 13]. The arthroscopic joint surface preparation technique spares local circulation and creates a more favorable environment for arthrodesis to occur, resulting in high fusion and low soft tissue complication rates. AAA reduces soft tissue disruption around the ankle joint, which may diminish the functional impairment of surrounding joints. We hypothesized that, after arthroscopic preparation of a joint surface, fixation performed with only two parallel, mediolateral HCSs provides adequate stability to achieve bone fusion [9-11]. The use of only two screws requires less talar and tibial surface, further facilitating bone fusion, minimizing the soft tissue damage from surgical approaches, and potentially decreasing the need for hardware removal [16, 17].

## MATERIALS AND METHODS

We retrospectively verified 24 consecutive patients (11 females, 13 males; 25 ankles) who underwent AAA stabilized with two parallel 7.3-mm HCSs introduced from the superomedial tibia into the inferolateral talus. The patients were treated between 2011 and 2015. The average follow-up was 26 months (range 18 to 52 months).

Arthrodesis was performed in 16 patients due to posttraumatic arthritis (in 5 as a sequela of pilon fractures, 6 ankle fractures, 3 spiral tibia fractures, and 2 had arthritis due to chronic instability after lateral ligament injury), in 4 patients due to neuromuscular ankle joint deformities (one myelomeningocele, one paralytic drop foot, and two post-polio cases), and in 4 patients due to rheumatoid arthritis. 3 patients with sequela of spiral tibia fractures, due to the obvious injury to the tibia, had overlooked and not treated syndesmotic injury with intact fibula in 2 and Maisonneuve fracture in 1 patient which created long standing instability and secondary osteoarthritis of the ankle joint.

Pre-operatively, all patients were independent ambulators, occasionally using walking aids as crutches. Bilateral fusion was carried out in one patient with bilateral post-pilon fracture arthritis. The mean age of the patients at the time of surgery was 54 years (range 18 to 73 years). In two patients with post-pilon fracture arthritis and two patients with post-tibia fracture arthritis, metal hardware from a previous surgery was removed during the index procedure. Patients with neuromuscular ankle deformity underwent additional procedures: percutaneous Achilles tenotomy (n=2) and tibialis posterior tendon transfer on the peroneus tertius tendon (n=1).

The mean angle of the pre-operative deformity of the ankle joint measured in weight-bearing anteroposterior and lateral radiograms was 10 degrees in the coronal plane – varus or valgus (range 0 to 15 degrees) and 20 degrees in the sagittal plane - plantar flexion (range 10 to 30 degrees). The highest sagittal deformity, up to 30 degrees, was observed in two post-polio patients. Achilles tendon percutaneous tenotomy was performed at the beginning of the surgical procedure when the equinus position of the foot was reduced to 10 degrees and the arthroscopic procedure initiated.

The primary objective of the present study was to evaluate the clinical effectiveness of AAA with stabilization with using two parallel mediolateral 7.3-mm HCSs in a heterogenic patient population involving posttraumatic, neuromuscular, and inflammatory arthritis. Because pain perception, activity level, use of walking aid, or use of shoe modifications are different in such a heterogenic group, no formal validated outcome measure could be used. The primary outcome was radiological and clinical fusion of the ankle joint. The secondary outcome was patient satisfaction with the performed procedure, which was measured as satisfied and would undergo the procedure again or not satisfied and would not undergo the procedure again.

Post-operative management consisted of 6 weeks of non-weight-bearing in a below knee cast, followed by full weight-bearing in a Walker boot until trabecular continuity was evident on control radiographs. In doubtful cases, a CT scan was used to confirm/exclude non-union. Patients received thromboprophylaxis with low-molecular-weight heparin during the full period of immobilization in the cast

### Surgical Technique

In the supine position, with an above knee tourniquet and sandbag under the ipsilateral buttock to achieve a perpendicular foot position, two standard arthroscopic (anteromedial and anterolateral) portals were utilized to prepare the joint for fusion. A 4.5 mm 30° scope, synovial shaver, oval acromionizer, small chisel, microfracturation chisels, and curettes were used. No joint distraction system was used. If present, anterior osteophytes were removed with thin osteotome and oval acromionizer. Remaining articular surface was removed with acromionizer and curette, to create healthy cancellous base. Articular surfaces of the medial malleolar gutters were removed. Lateral gutter was meticulously cleared to allow proper joint positioning and compression. Curved microfracturation chisel was then used to, especially in posterior part of the joint, to enlarge bleeding surface. After joint surface preparation, the foot was positioned correctly in neutral flexion, from 0° up to 5° of hindfoot valgus and external rotation from 5° to 10°. Following joint surface preparation three steps are taken for screw fixation. Following joint surface preparation three steps are taken for screw fixation.

Step 1: Retrograde 2.5-mm Kirschner wire (K-wire) introduction into the lateral talar process

**Fig. (1):**
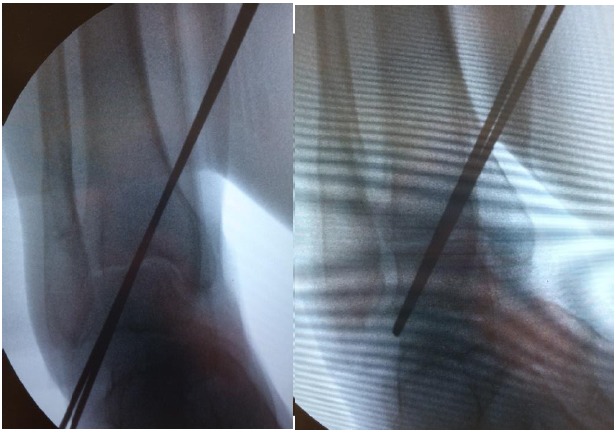
C-arm view of the right ankle with two parallel K-wires introduced from lateral talar process across debrided ankle joint in Step 1 of the procedure (left). K-wires are then withdrawn with the power drill on the medial side, until they are completely flush with the lateral talar process, what can be checked by a finger palpation and/or C-arm control in Step 2 (right).

The lateral talar process is an easily palpated structure, even in cases with severely distorted ankle anatomy. A small 1-2 cm approach is created over the sinus tarsi following a relaxed skin tension crease. When the correct foot alignment was held manually by an assistant, two parallel K-wires (2.5 mm diameter, 230 mm length) were introduced into the inferolateral part of the lateral talar process and directed upward and slightly posterior to enter the medial tibial cortex. C-arm control in two perpendicular planes is mandatory (Fig. **1**). The whole area, from the posterior edge of the lateral talar process to the base of the talar neck, was available to introduce the K-wire. Peroneal tendons must be protected with a blunt retractor and care must be taken to pull tendons away during K-wire introduction and passage. The sharp K-wire tip easily penetrates the softer bone of the lateral talar process, which facilitates its further introduction and advancement control.

Step 2. Antegrade (medial tibia) K-wire withdrawal and measurement screw length

**Fig. (2):**
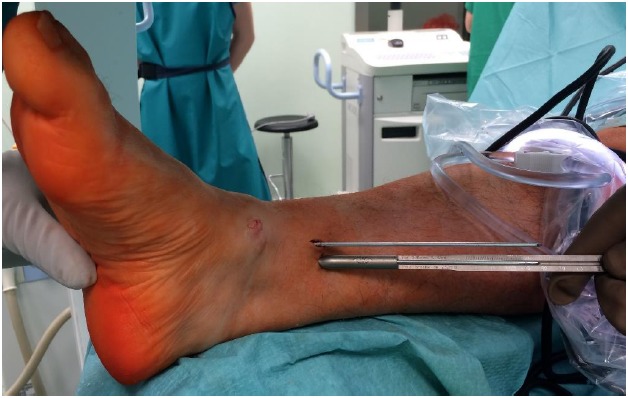
Precise measurement of the screw length. The K-wires were withdrawn on the medial tibia until they were completely flush with the lateral talar process, which can be checked by finger palpation and/or C-arm control. This step allows for very precise measurement of the screw length over the K -wire.

The K-wires were withdrawn with the power drill on the opposite side of the medial tibia until they were completely flush with the lateral talar process, which can be checked by finger palpation and/or C-arm control (Fig. **1**). This step allows for very precise measurement of the screw length over the K -wire. After measuring the screw length over the K-wire (Fig. **2**), the K-wires were pushed back with the power drill again to stick out from the talus entry points. This maneuver serves as a backup and helps remove K-wires in case they break or bend while drilling with a cannulated drill or during final screw insertion.

Step 3. Antegrade drilling and screw insertion

**Fig. (3):**
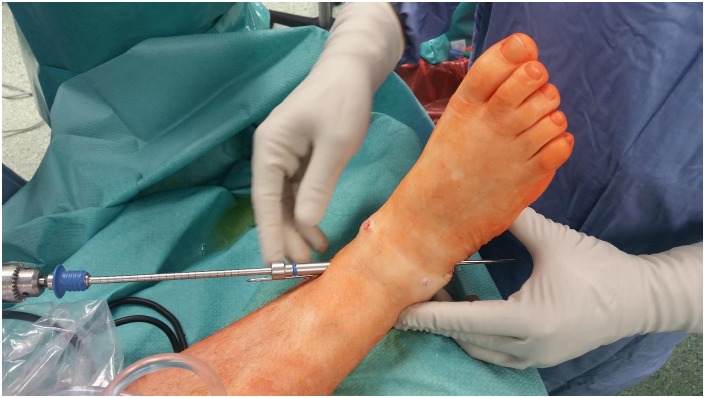
Antegrade drilling with soft tissue protector inserted. Note the K-wires are pushed back down again and extend from wound on the lateral side. That manoeuvre serves as a backup and helps to remove K-wires in case they brake or bent while using cannulated drill.

**Fig. (4):**
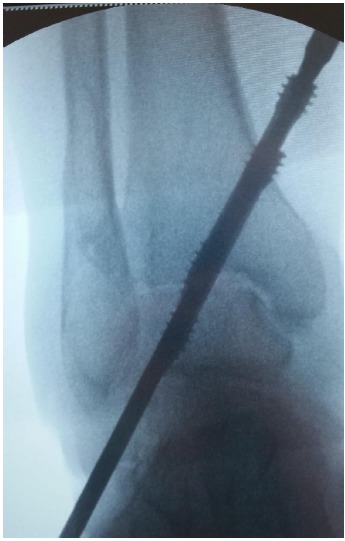
C-arm control while inserting second HCS. Both screws are tightened and buried inside medial tibial cortex.

**Fig. (5):**
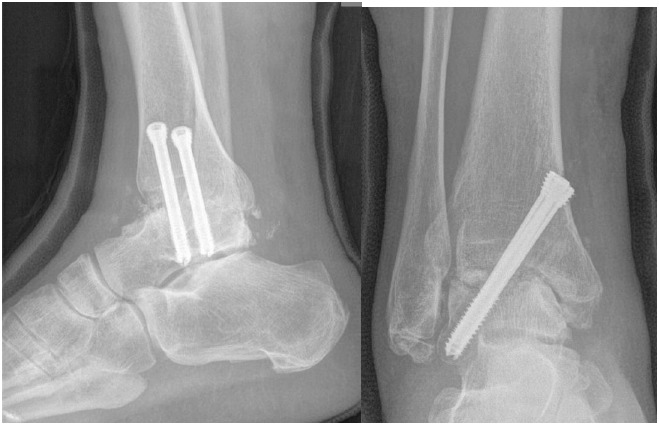
Non-weight bearing AP and lateral radiographs two weeks after AAA with two parallel HCS compression. Note distal HCS threads are within lateral talar process and proximal covered in thinner medial tibial cortex causing any impingement on soft tissues.

**Fig. (6):**
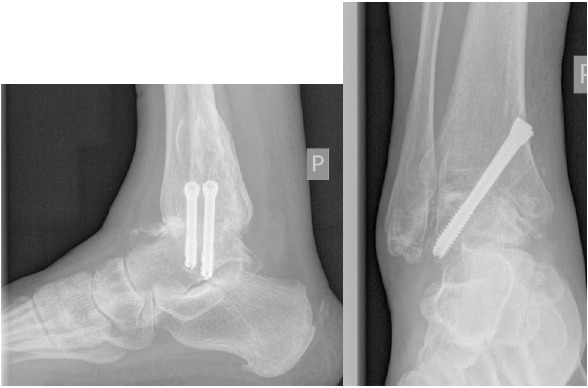
Weight bearing AP and lateral radiographs six weeks after AAA with two parallel HCS compression. Note radiographic bone union at the arthrodesis site.

A stab incision was performed around the K-wires to the medial tibia, a soft tissue protector inserted, and cannulated drill introduced from the tibial medial cortex into the talus. A 7.3-mm cannulated HCS was introduced to the point that the entire head was inside the cortex (Fig. **3**). Two HCSs were used in every patient (Figs. **4**-**6**).

## RESULTS

Fusion occurred in 23 joints (92%) over an average of 12 weeks (range 6 to 18 weeks). Ankle arthrodesis was not achieved in 2 joints (8%), both in post-pilon fracture patients, and they underwent subsequent revision surgery 12 months after the index procedure. The correct foot alignment (neutral flexion and from 0° up to 5° of hindfoot valgus) was not achieved in 4 feet (16%). Two feet remained in residual equinus (10 degrees) and 2 in varus (10 degrees) deformity. Two residual equinus deformities persisted in post-pilon fracture patients. Residual varus malalignment persisted in a patient with persistent talar tilt in chronic lateral instability arthritis and one patient with paralytic drop foot. None of the patients with malunion agreed with further revision surgery. One patient with bilateral ankle fusion developed symptomatic arthritis in one subtalar joint during the follow-up period and was treated with pantalar fusion. No wound infection or thromboembolic complications were noted during the follow-up period.

Twenty patients (83%) were satisfied with the ankle fusion and would undergo the procedure again. Four patients were not satisfied and would not undergo ankle fusion again, two because of non-union, one with early subtalar arthritis, and one with residual varus malalignment. Three out of four dissatisfied patients belonged to the post-pilon fracture group. None of the patients required screw removal for prominence during the follow-up period.

## DISCUSSION

Ankle arthrodesis remains the procedure of choice to reduce pain and recreate plantigrade foot in patients with end-stage ankle arthritis and deformity caused by a spectrum of diseases [1-3]. Ankle arthrodesis is increasingly performed with arthroscopic assistance [5, 6]. Uncomplicated fixation methods to maintain proper foot alignment long enough to achieve solid bone union is a major concern of many orthopedic surgeons [9, 10, 12-16]. The ideal technique for obtaining adequate fusion is based on the least amount of hardware to achieve the strongest mechanical stability while preserving the biological environment for fusion to occur. The number of screws necessary to achieve solid fusion varies between studies. In a sawbones model study, Brodsky *et al.* found that the addition of a third screw does not result in a major decrease in the amount of bone contact available for fusion [16]. In a composite ankle joint model, Clifford *et al.* found no significant differences in bending stiffness between anterior plate or lateral plate fixation and the three compression screws construct, suggesting that the addition of a compression screw will considerably increase the primary bending stiffness of ankle arthrodesis [14]. Yoshimura *et al.* found that the fastest bone union in AAA is achieved with three parallel 6.0-mm cannulated cancellous screws placed medially from the distal tibia into the talus [16]. Arthroscopic joint preparation creates a more favorable environment for fusion and decreases the number of screws used [2, 6]. Vaughan *et al.* presented the results of bilateral ankle arthrodesis via a mini open technique and fixation with only two cancellous screws, achieving a 100% fusion rate [19]. In the largest published AAA series to date, Winson *et al.* used only two parallel 6.5-mm AO screws placed medially from tibia into the talus, achieving bone union in 92% of cases after an average of 12 weeks. The important complication mentioned by the authors was prominent hardware that had to be removed in 21% of patients [8]. Danawi *et al.* achieved a 91% fusion rate using two parallel compression screws but reported problems with a subsequent need for prominent hardware removal [3]. Townshend reported the removal of symptomatic implants only in the arthroscopic group when comparing open and arthroscopic ankle arthrodesis [2]. Thus, open techniques may allow for more precise screw length measurement than arthroscopic techniques. HCS introduction seems to resolve the problems of easier fixation, inter-fragmentary compression, and need for hardware removal. Using only two Acutrak 6/7-mm HCSs, Odutola *et al.* achieved 88% bony fusion and no complications or pain caused by metalwork [9]. In a cadaver model, Somberg *et al.* found no difference in biomechanical stability between three Acutrak HCSs and three traditional screws [10]. Jehan and O’Hill presented a technique for parallel compression screw fixation enhanced with autologous bone graft, even in difficult cases after failed ankle arthroplasty [18]. Kennedy *et al.* confirmed that a simple two-screw technique supported with biological and mechanical principles can achieve excellent fusion rates [15]. Jain *et al.*, using two-screw stabilization and an arthroscopic technique in a series of 52 fused ankles, achieved a fusion rate similar to the present study (92%) [19]. They reported delayed union in a post-pilon fracture patient. Two non-union cases from the present paper were also sequelae of pilon fracture. Another post-pilon fracture patient developed malunion in the equinus position. It is not unusual to find bone defects in the tibial plafond following pilon fracture and its surgical treatment [6]. Patients with ankle arthritis secondary to pilon fracture may require more careful, detailed pre-operative assessment of the tibia bone stock with CT imaging and open fusion using bone grafts. Jain *et al.* distinguished two other factors potentially leading to non-union: smoking and neuromuscular disorders. However, all neuromuscular patients in the present study achieved solid fusion up to 12 weeks. All neuromuscular patients underwent additional soft tissue balancing procedures, which helped reduce the constant pathological overpull from contracture muscles. None of the treated patients were active smokers because they were strongly advised to refrain from smoking 3 months before surgery, otherwise patients were not qualified for surgery.

The main limitations of the present study are the lack of a control group and the lack of a valid outcome measure. Thus, functional results cannot be compared with other techniques and studies. However, the very low non-union rate is comparable to other ankle arthroscopic and open techniques [1-4, 12, 14, 19, 20].

## CONCLUSION

The presented technique was successful in achieving a high fusion rate in a variety of diseases, decreasing intra- and post-operative hardware complications while maintaining temporary and final bone stability. Patients with ankle arthritis secondary to pilon fracture may require accessory assessment of the tibial bone stock prior to arthrodesis and, in doubtful cases, bone grafting with more rigid stability to achieve ankle fusion
